# Vaginal Extrusion of Transobturator Tape 6 Years after Procedure: How Long Should Be the Follow-Up Period after Vaginal Sling Surgeries?

**DOI:** 10.1155/2012/527345

**Published:** 2012-11-12

**Authors:** Chanchal Singh, Roy Ng Kwok Weng

**Affiliations:** ^1^Department of Obstetrics and Gynaecology, National University Hospital, 5 Lower Kent Ridge Road, Singapore 119074; ^2^Division of Urogynaecology, Department of Obstetrics and Gynaecology, National University Hospital, Singapore 119074

## Abstract

A 77-year-old lady presented to the gynaecology outpatient clinic with vaginal extrusion (3C/T3/S1) six years after a transobturator suburethral sling procedure (TOT). The entire tape was spontaneously extruded vaginally and the vaginal erosion healed with conservative management. Midurethral tapes, currently the standard of care in the management of urodynamic stress incontinence (USI), are associated with a risk of erosion and extrusion of the synthetic material. The present case highlights the importance of the long-term followup after any sling procedure as erosion and/or extrusion may arise at any time following the procedure.

## 1. Introduction 

Midurethral tapes are now considered the standard of care in management of urodynamic stress incontinence (USI). These are minimally invasive, short duration procedures with minimal complications in experienced hands and comparable efficacy to other surgical procedures. The transobturator “outside-in” approach was first performed by Delorme in 2001 and is associated with fewer complications than retropubic slings with similar subjective cure rates [1]. However this approach is associated with its own unique complications. It has also been found to be associated with higher rates of vaginal erosions [2]. Most complications present within 6 weeks to 3 months of the procedure. There are no definite guidelines as to how long the followup should be after these procedures. 

## 2. Case 

**Figure 1:**
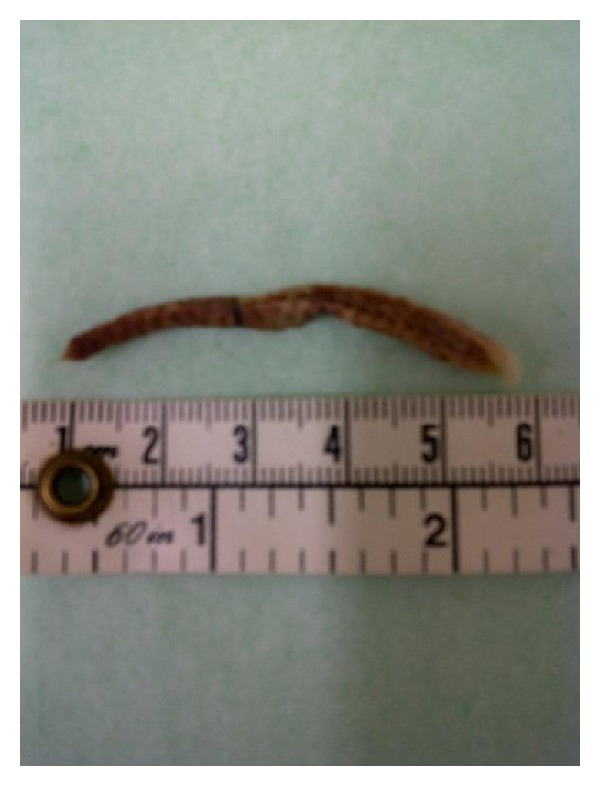
The spontaneously extruded transobturator tape (TOT).

A 77-year-old lady presented to the outpatient clinic with a complaint of foul smelling vaginal discharge for 4 weeks. She had no fever, abdominal or thigh pain, difficulty in walking, or postmenopausal bleeding. She had undergone vaginal hysterectomy with pelvic floor repair and transobturator tape insertion (TOT) for third degree uterovaginal prolapse and USI 6 years ago at our institution. The tape used was Obtape (Mentor-Porges, Le Plessis-Robinson, France). She had no hesitancy, stress, or urge incontinence and was able to empty the bladder completely at one-year followup after surgery. She defaulted further followup but had been asymptomatic until current presentation. She was sexually inactive for the last 8 years. She had no history of diabetes. Her BMI was 22. General physical examination was unremarkable and abdomen was soft. There was no evidence of sepsis or cellulitis. There was no cystocele, rectocele, enterocele, or vault prolapse. On speculum examination there was foul smelling discharge. A light green braided suture about 2-3 cm was seen protruding from the middle of the vault surrounded by granulation tissue. The suture was held with an artery forceps and cut at its the base. This was a loop remnant of Ethibond number 1 suture used for McCall's culdoplasty during vaginal hysterectomy. On vaginal examination, a 4 to 5 cm tape segment was felt protruding through the vagina in the midurethral region. During the digital examination of the tape extrusion, it literally came off the vagina (Figure 1). There was no tenderness on examination. A vaginal swab was sent for culture and antibiotic pessary was prescribed for 7 days. Urine microscopy and culture were negative. She was reviewed in the clinic 2 weeks and 6 weeks later. Her discharge resolved completely. She continues to be asymptomatic and has no complaint of urinary incontinence. The vaginal swab culture was reported as mixed bacterial growth. 

## 3. Discussion 

The transobturator approach, wherein the tape traverses the obturator fossa and exits through skin at the groin area, is associated with lower rates of voiding dysfunction, bladder injury, and blood loss as compared to retropubic slings [1]. Rarely, it may lead to potentially serious complications like obturator abscess, obturator haematoma, perineal cellulitis, and abscess formation in the adductor group of muscles [2–4]. A major concern with the use of synthetic slings is erosion of the tape. The reported rates of intravaginal erosion of suburethral slings range from 0% to 13.8%. The rates are higher with nonknitted polypropylene tapes like Uratape (type IV) or Obtape (type II) as compared to knitted macroporous monofilament (type I) tapes (<1%) used in TVT and TVT-O [2]. It is likely that this incidence is underreported as small erosions are known to heal spontaneously. 

When vaginal erosion occurs, the patient usually presents with persistent vaginal discharge, vaginal bleeding, postcoital bleeding, or male partner discomfort or pain during intercourse. Diagnosis is confirmed by visual inspection or palpation of the tape in the vagina. Care must be taken to exclude urethral and bladder erosions that are potentially serious complications and necessitate immediate surgical removal. As per the International Urogynecological Association (IUGA)/International Continence Society (ICS) joint terminology and classification, the complication in our patient would be classified as 3C/T3/S1 [5]. 

The reported mean time for presentation of symptoms caused by vaginal erosion of the tape after TOT procedure is 9 months [3]. Our patient's presentation is unusual as she presented 6 years after the procedure thus highlighting the need for a long-term followup after midurethral sling procedures. The proposed mechanism of vaginal erosion is subclinical infection of the foreign body leading to erosion of the overlying vaginal mucosa. The age related atrophic vaginitis might have contributed to the late presentation in this case. The tape extrusion could have occurred earlier as the patient did not return for a followup after being seen at 1 year until the current presentation, 6 years postoperatively. 

Management depends on the site of erosion, its extent, severity of symptoms, and the type of tape used. Vaginal erosions are associated with less morbidity as compared to urethral or bladder erosions. Most authors recommend surgical removal of the mesh [3] especially if it is not a type I tape, that is, if it is a nonwoven thermally bonded polypropylene mesh like Uratape (type IV) or Obtape (type II). Such tape erosions should be treated by immediate and maximal tape removal to prevent life-threatening infections like obturator abscess and perineal cellulitis [2–4]. If there is extrusion of a type I tape in the lateral vaginal sulcus causing dyspareunia, it can be excised in the outpatient setting under local anaesthesia. An asymptomatic tape extrusion in an elderly lady who is not sexually active may be treated conservatively. Vaginal oestrogen may be prescribed in case of atrophic vaginitis. If there are signs of infection, vaginal or oral antibiotics should be prescribed. Partial removal of only the extruded part of a type I tape may prevent recurrence of stress urinary incontinence. 

The present case highlights the importance of a long-term followup after any sling procedure as erosion may arise at any time following the procedure. We recommend that the followup after the procedure should be at 1 week, 4 to 6 weeks, 6 months, and yearly thereafter for at least 5 years as required if symptomatic of tape extrusion, failed surgery, overactive bladder, or voiding difficulty.
